# Renal Function and Serum Neurofilament Light Chain in Acute Ischemic Stroke: An Observational Cohort Study

**DOI:** 10.3390/diagnostics16132113

**Published:** 2026-07-06

**Authors:** Federica Ferrari, Nicola Davide Loizzo, Federico Mazzacane, Beatrice Del Bello, Salvatore Console, Silvia Scaranzin, Chiara Morandi, Matteo Gastaldi, Alessandra Persico, Anna Cavallini

**Affiliations:** 1 Department of Health Sciences, Università del Piemonte Orientale, Via Solaroli 17, 28100 Novara, Italy; federica.ferrari@uniupo.it; 2 Department of Brain and Behavioral Sciences, University of Pavia, Viale Golgi 19, 27100 Pavia, Italy; 3 Department of Stroke Unit and Emergency Neurology, IRCCS Mondino Foundation, Via Mondino 2, 27100 Pavia, Italy; nicola.loizzo@mondino.it (N.D.L.);; 4 Neuroimmunology Research Section, IRCCS Mondino Foundation, Via Mondino 2, 27100 Pavia, Italy

**Keywords:** acute ischemic stroke, neurofilament light chain, serum biomarkers, renal function, estimated glomerular filtration rate, chronic kidney disease, prognosis, functional outcome, neuroaxonal injury, Ella^TM^

## Abstract

**Background/Objectives**. Neurofilament light chain (NfL) is a biomarker of axonal injury with prognostic value in acute ischemic stroke and a promising surrogate outcome marker. This study evaluated whether serum NfL concentrations in ischemic stroke were modified by varying degrees of renal function. **Methods**. In this prospective, single-center observational study, patients aged 18–80 y admitted to the IRCCS Mondino Foundation—Stroke Unit between May 2022 and August 2024 were enrolled. Inclusion criteria were: radiologically confirmed ischemic stroke within 24 h of onset, NIHSS ≥ 1 at admission, pre-stroke mRS < 2, no other neurological comorbidities, and eGFR > 30 mL/min/1.73 m^2^. Serum creatinine was measured on admission, and eGFR was calculated using the CKD-EPI equation. Serum NfL was measured by Ella™ immunoassay at T0 (≤24 h), T1 (5 ± 3 d), and T2 (7 ± 3 d). Factors associated with serum NfL concentrations were assessed using linear mixed-effects models, and prognostic associations were evaluated by multivariate logistic regression. **Results**. Ninety-seven patients were included (median age 68.3 y; 39.2% female). Higher NfL levels were independently associated with lower eGFR (−2.4% per mL/min/1.73 m^2^ increase; 95% CI −3.2% to −1.6%; *p* < 0.001), and higher NIHSS at admission (+3.5% per point; 95% CI 0.7% to 6.4%; *p* = 0.014). Time from stroke onset was also associated with NfL (*p* < 0.001). Among patients with 3-month follow-up and T2 measurement (*n* = 62), the main effects of log10-transformed NfL at T2 and eGFR were not independently associated with unfavorable outcomes. However, a significant log10 NfL × eGFR interaction was observed (OR 0.22; 95% CI 0.07–0.73; *p* = 0.014), indicating that the prognostic association of NfL varied according to renal function. **Conclusions**. Renal function affects serum NfL after ischemic stroke and appears to modify its prognostic association with 3-month outcomes.

## 1. Introduction

Stroke is one of the leading causes of mortality and long-term disability worldwide, and identifying reliable prognostic biomarkers to guide clinical decision-making represents a major unmet need in stroke research. Neurofilament light chain (NfL) is a structural component specific to the neuronal cytoskeleton, essential for maintaining axonal integrity and function [1]. The development of ultra-sensitive assay technologies has made NfL measurements feasible in easily accessible blood samples. As a result, NfL is increasingly used as a minimally invasive surrogate marker of neuronal damage across a broad spectrum of neurological conditions. In chronic neurodegenerative diseases such as Alzheimer’s disease, amyotrophic lateral sclerosis, and multiple sclerosis, circulating NfL concentrations reflect the cumulative burden of axonal injury and are increasingly adopted as surrogate endpoints in clinical trials [2]. In the acute setting of cerebral ischemia, elevated serum NfL levels have been shown to correlate with stroke severity and long-term functional outcomes, positioning NfL as a promising prognostic tool [3,4,5].

However, serum NfL concentrations can be influenced by confounding factors. Therefore, identifying and characterizing these determinants is crucial for the correct interpretation of NfL measurements. Impaired renal function has emerged as one of the most relevant factors. An inverse correlation between serum NfL concentrations and kidney function has been documented in healthy individuals and in patients with chronic neurological diseases, likely reflecting reduced renal clearance of neurofilament proteins [6,7,8].

Critically, all studies characterizing the renal–NfL relationship to date have been conducted in healthy subjects, during physiological aging, or in chronic neurodegenerative conditions. No study has specifically examined this relationship in the acute context of ischemic stroke (IS). This distinction is biologically significant: IS triggers a massive and sudden release of NfL into the bloodstream, in sharp contrast to the slow, gradual release observed in neurodegenerative diseases. This fundamentally different kinetic profile could alter the influence of renal function on NfL clearance and circulating concentrations. Therefore, dedicated studies in patients with ischemic stroke are warranted.

The aim of this study was to evaluate serum NfL concentrations, assayed by Ella™ automated immunoassay system, in IS patients with varying degrees of renal function.

## 2. Materials and Methods

### 2.1. Patients’ Population

Patients with acute ischemic stroke admitted to the Stroke Unit of IRCCS Mondino Foundation between May 2022 and August 2024 were screened for eligibility. A stroke diagnosis was formulated in patients with a sudden onset of neurological clinical symptoms and signs, and a consistent acute ischemic lesion confirmed by neuroimaging (head CT or MRI).

Patients of both sexes with the following characteristics were included: age ≥ 18 years, within 24 h from neurological symptom onset, with ischemic stroke confirmed at brain CT and/or MRI scan, a neurological deficit in the Emergency Department (ED) scored via the National Institute of Health Stroke Scale (NIHSS) ≥ 1, and a pre-stroke functional disability assessed by the modified Rankin Scale (mRS) scored 0–1. Exclusion criteria were: age > 80 years, transient ischemic attack, previous clinically symptomatic ischemic and/or hemorrhagic stroke, previous traumatic head injuries, active central or peripheral nervous system disease other than the index stroke event, renal insufficiency with an estimated Glomerular Filtration Rate (eGFR) < 30 mL/min, severe hepatic insufficiency (Child-Pugh B or C; or ALT and/or AST > 2.5× the upper normal values), active oncological disease, life expectancy < 12 months for non-neurological causes, and pregnancy. Patients were treated as per the standard of care according to the latest European guidelines. Stroke etiology was classified according to the TOAST classification [9].

The study was conducted according to the STROBE statement (Strengthening the Reporting of Observational Studies in Epidemiology) [10].

### 2.2. Clinical Variables and Laboratory Data

The following variables were collected prospectively and included in an ad-hoc database: (1) patient demographics: age, sex, ethnicity, weight, height and body mass index (BMI) [11]; (2) vascular risk factors: arterial hypertension, atrial fibrillation, diabetes mellitus, dyslipidemia, smoke exposure, carotid atherosclerotic disease, previous acute coronary syndrome, and valvular heart disease; (3) medical therapy: antiplatelets, anticoagulants, and lipid-lowering drug use before the index event; (4) neurological deficit at onset measured by NIHSS [12], pre-stroke mRS [13], and reperfusion therapies.

The following laboratory data were also collected upon admission to the Stroke Unit: blood cell count, total cholesterol, HDL cholesterol, LDL cholesterol, triglycerides, glycemia, glycated hemoglobin (HbA1c), and C-reactive protein. Renal function was assessed by serum creatinine, from which the eGFR was derived using the Chronic Kidney Disease—Epidemiology Collaboration (CKD-EPI) formula, as follows:

$$eGFR=\;141\times (\frac{Scr}{\kappa },1{)}^{\alpha }\times (\frac{Scr}{\kappa },1{)}^{-1.209}\times 0.993^{Age}\times 1.018[if\;female]\times 1.159[if\;black]$$

where *Scr* is serum creatinine, *κ* is 0.7 for females and 0.9 for males, *α* is −0.329 for females and −0.411 for males, min indicates the minimum of *Scr*/*κ* or 1, and max indicates the maximum of *Scr*/*κ* or 1 [14]. Classes of chronic kidney disease were attributed according to the Kidney Disease: Improving Global Outcomes (KDIGO) classification [15]: specifically, renal function was classified as normal or high (G1) for eGFR ≥ 90 mL/min/1.73 m^2^, mildly reduced (G2) for eGFR 60–89 mL/min/1.73 m^2^, mildly to moderately reduced (G3a) for eGFR 45–59 mL/min/1.73 m^2^, and moderately to severely reduced (G3b) for eGFR 30–44 mL/min/1.73 m^2^. As previously stated, patients with eGFR < 30 mL/min/1.73 m^2^ were excluded by study design.

### 2.3. Serum Sample Collection and NfL Assay

Venous blood samples (15 mL) were collected in vacutainers with clot activator from all patients upon enrollment in the study within 24 h of symptom onset (T0). Additional samples were obtained at 5 ± 3 days (T1), and 7 ± 3 days (T2), after stroke (with a minimum 24 h interval from the previous one) if the patients were still admitted to our Stroke Unit. These sampling time points were selected according to the established temporal profile of serum NfL after ischemic stroke, with T0 representing the hyperacute phase, T1 the period of progressive NfL increase, and T2 approximating the expected peak concentration during the first week after stroke. Previous longitudinal studies have shown that serum NfL levels rise progressively following acute ischemic stroke and reach their highest concentrations approximately 7–10 days after symptom onset, when the association with infarct volume and functional outcome is strongest [4,16,17]. Within two hours of collection, the blood samples were centrifuged at 4000 rpm for 15 min at room temperature to obtain serum, which was aliquoted into cryovials and stored at −80 °C until use.

A Human NfL Simple Plex assay (ProteinSimple, Minneapolis, MN, USA) was employed on an Ella™ automated immunoassay system (ProteinSimple, Minneapolis, MN, USA), according to the manufacturers’ instructions. Calibration of Ella™ was performed using the in-cartridge factory standard curve, and serum samples were measured with a 1:2 dilution in Sample Diluent (ProteinSimple, Minneapolis, MN, USA). A single well was used for each serum sample, as triplicate assays are automatically performed in the Simple Plex assay microfluidic platform. The NfL lower limit of quantification (LLoQ) is 2.70 pg/mL, the upper limit of quantification (ULoQ) is 10,290.00 pg/mL, and the limit of detection (LOD) is 1.09 pg/mL.

### 2.4. Statistical Analysis

Continuous variables with a normal distribution were summarized using means and standard deviations, while those with a non-normal distribution were reported as medians and interquartile ranges (IQR). Normal distribution was assessed by the Shapiro–Wilk test. Categorical variables were presented as counts and percentages.

Serum NfL concentrations were log10-transformed before correlation and inferential analyses to improve normality and stabilize variance. 

Correlations between eGFR and serum NfL concentrations at each time point were assessed using Pearson correlation coefficients. 

Changes in serum NfL levels across the three time points were assessed using linear mixed-effects models, accounting for repeated measurements within individuals. No formal imputation of missing data was performed; missing repeated NfL measurements were handled within the linear mixed-effects model framework, under the missing-at-random assumption. Time from stroke onset was modeled as a categorical variable, with T0 as the reference. Renal function, expressed as eGFR calculated using the CKD-EPI equation, was included as a predefined covariate of interest. The linearity assumption for continuous predictors on the log10-NfL scale was assessed during model building. No relevant departure from linearity was observed for eGFR, which was therefore retained as a continuous linear variable. 

Given the observational design of the study, additional clinical variables were evaluated as factors potentially associated with serum NfL levels rather than causal predictors. Candidate covariates were selected based on statistical association, clinical relevance, and their plausible biological relationship with renal function/eGFR. Variables selected according to these criteria were entered into the multivariable model. Specifically, BMI and pre-stroke mRS were forced into the models. In fact, the inverse relationship between BMI and NfL was previously reported in a study involving both multiple sclerosis patients and healthy controls [18], likely because individuals with higher BMI may have greater blood volume, leading to a dilutional effect and consequently lower measurable NfL concentrations. Similarly, a proportional relationship has been described between NfL concentrations and functional dependency assessed by mRS [19].

As a secondary clinically oriented analysis, renal function was also analyzed according to KDIGO CKD stages. Because of the limited number of patients in G3a and G3b categories, these stages were combined into a single G3 category for inferential analyses. CKD stage was then entered as a categorical predictor in the linear mixed-effects model, with G1 as the reference category, adjusting for time from stroke onset and NIHSS at admission.

Model coefficients for log10-transformed NfL were back-transformed to express results as percentage changes in absolute NfL concentrations, using the formula (10*β* − 1) × 100, where *β* represents the estimated regression coefficient.

To evaluate whether renal function modified the prognostic association between serum NfL and 3-month functional outcome, a multivariable logistic regression model was fitted as a complete-case analysis among patients with available T2 NfL measurement and 3-month follow-up. The T2 time point, corresponding to 7 ± 3 days from stroke onset, was selected based on evidence indicating that NfL levels increase during the first week after ischemic stroke and that measurements obtained several days after the acute event show more consistent and stronger associations with functional outcome compared with very early sampling [5].

The dependent variable was unfavorable functional outcome, defined as a modified Rankin Scale of score 3–6 at 3 months. The model included log10-transformed NfL at T2, baseline eGFR, their interaction term, age, and baseline NIHSS. Both log10-transformed NfL and eGFR were mean-centered before calculation of the interaction term.

Pre-stroke mRS was not included in the multivariable model because of its very limited variability in the study population. Odds ratios (ORs) with corresponding 95% confidence intervals and *p*-values were reported.

To improve the interpretability of the log10 NfL × eGFR interaction term, post-estimation predicted probabilities of unfavorable 3-month outcome were calculated across representative serum NfL values and clinically meaningful eGFR strata. Predicted probabilities were estimated from the revised multivariable logistic regression model including log10-transformed serum NfL at T2, eGFR rescaled per 10 mL/min/1.73 m^2^, the log10 NfL × eGFR interaction term, age, and NIHSS at admission. Pseudo-R^2^ values were reported as descriptive measures of model fit.

To assess whether serum NfL added prognostic information beyond standard clinical predictors, nested logistic regression models were compared using likelihood-ratio tests: a base model including age, baseline NIHSS, and eGFR was compared with a model additionally including log10-transformed NfL at T2, and with the full model including log10-transformed NfL, eGFR, and their interaction term.

Exploratory ROC curves and AUC values were calculated in the complete-case subgroup to assess discrimination for unfavorable 3-month functional outcome, both for serum NfL at T2 alone and for the multivariable prognostic models. AUCs were compared using ROC curve comparison tests.

A preliminary sample size estimation was performed based on literature data from biomarker studies using functional outcome (mRS scores) as an endpoint, although not specifically tailored to the present primary analyses focused on renal function. As the study was observational, the final sample size was ultimately determined by feasibility and the recruitment period rather than a formal power calculation for the outcomes evaluated here.

All statistical analyses were performed using Stata BE (Basic Edition) version 17.0; graphics were made with R version 4.4.2. A two-sided *p*-value < 0.05 was considered statistically significant.

## 3. Results

Ninety-seven patients were included in the study. The median age was 68.3 years (IQR 15.4), and 38 (39%) were female. The median NIHSS score at initial evaluation was 5 (IQR 7), and 1 at discharge (IQR 3). The median pre-stroke disability score was 0 (IQR 0). Patients’ characteristics are summarized in Table 1.

According to KDIGO categories, renal function was found to be normal (G1) in 28 patients (28.9%), mildly reduced (G2) in 53 (54.6%), mildly to moderately reduced (G3a) in 9 (9.3%), and moderately to severely reduced (G3b) in 7 patients (7.2%).

### 3.1. Serum NfL Concentrations over Time

All patients had NfL values available at T0, while T1 and T2 blood samples were collected in 86 (89%) and 68 (70%) of patients, respectively. Loss of samples at T1/T2 mainly reflected early discharge or logistical reasons. The analysis included a total of 251 observations from 97 patients.

**Figure 1:**
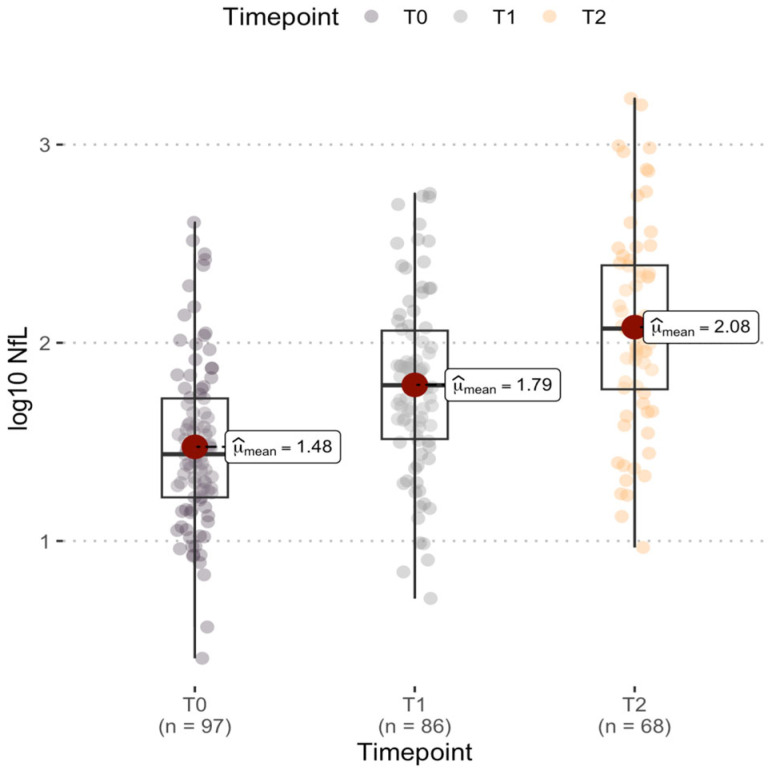
Log10-transformed NfL serum concentrations after 24 h from stroke onset (T0), after 5 ± 3 days (T1) and after 7 ± 3 days (T2).

An increase in median NfL levels was observed over time from stroke onset: 27.4 pg/mL (IQR 35.8) at T0, 61.1 pg/mL (IQR 84.8) at T1, and 118 pg/mL (IQR 190.6) at T2. This temporal trend was confirmed by the linear mixed-effects model with log10-transformed NfL as the dependent variable and time modeled as a categorical fixed effect. NfL concentrations were significantly higher at both T1 and T2 compared with T0 (overall *p* < 0.001). Specifically, in the unadjusted time-effect model, compared with baseline, NfL levels increased by 115% (95% CI 90–143%; *p* < 0.001) at T1 and by 267% (95% CI 211–333%; *p* < 0.001) at T2, confirming a marked rise during the first week after stroke (Figure 1).

### 3.2. Correlations Between Serum NfL Levels and Renal Function

**Figure 2:**
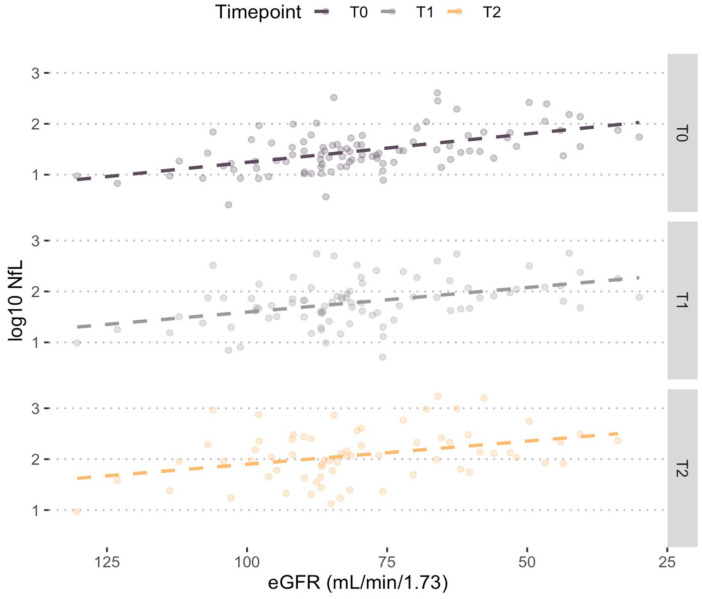
Correlations between NfL serum concentrations expressed as (Log10) and eGFR at the considered time-points.

The correlation analysis between serum neurofilament concentrations and renal function revealed a consistent inverse linear relationship across all three observation time points. This correlation was strongest at T0 (r = −0.53; *p* < 0.001), followed by T1 (r = −0.43; *p* < 0.001) and T2 (r = −0.35; *p* = 0.003). These findings are visually represented in Figure 2.

### 3.3. Factors Associated with Serum NfL Concentrations

Consistent with the study hypothesis, time from the index event and renal function, included as predefined fixed effects, were significantly associated with serum NfL concentrations in the mixed-effects model (*p* < 0.001 for both). Further variables found to be significantly associated with NfL in the univariate analysis were, notably, as follows: age at time of neurological event (*p* < 0.001), NIHSS at admission (*p* = 0.006), smoking (*p* = 0.059), hypertension (*p* = 0.01), carotid atherosclerotic disease (*p* = 0.006) and HDL cholesterol (*p* = 0.006). Body mass index (*p* = 0.583) and pre-stroke functional status (*p* = 0.586) were not associated with NfL values (Table 2), but they were forced into the models based on their biological plausibility, as discussed previously in Section 2.4. Therefore, the variables associated with NfL values at the univariate analysis were incorporated into the multivariable model, along with BMI and pre-stroke mRS.

**Table 2:** Univariate analysis performed using a linear mixed-effects model (REML) with an exchangeable residual variance structure. Dependent variable: log10-transformed neurofilament concentration.

Variable	Coefficient	95% CI	*p*-Value
Age	0.0149179	[0.0091084; 0.0207274]	<0.001
BMI	−0.0054274	[−0.024824; 0.0139693]	0.5834 ^1^
Pre-stroke mRS	0.0546836	[−0.1419783; 0.2513456]	0.5858 ^1^
NIHSS in ED	0.0194409	[0.0055571; 0.0333247]	0.0061
Hypertension	0.2081227	[0.0479903; 0.3682551]	0.0109
Atrial Fibrillation	0.3141259	[0.0724081; 0.5558437]	0.0109
Diabetes	0.1461684	[−0.0950109; 0.3873478]	0.2349
Dyslipidemia	0.1391916	[−0.0327; 0.3110832]	0.1125
Smoking	0.156462	[−0.0061984; 0.3191224]	0.0594
Carotid atherosclerotic disease	0.2876395	[0.0834581; 0.491821]	0.0058
Coronary artery disease	0.1582197	[−0.1824378; 0.4988773]	0.3627
Valvular heart disease	0.2023325	[−0.0158381; 0.4205031]	0.0691
HDL cholesterol	−0.0081038	[−0.013912; −0.0022957]	0.0062

^1^ Variable not statistically significant but retained in the final model due to biological plausibility. Abbreviations: BMI, body mass index; ED, Emergency Department; NIHSS, National Institute of Health Stroke Scale; mRS, modified Rankin Scale.

After stepwise backward elimination, the final multivariate model identified three factors independently associated with serum NfL concentration: lower estimated renal function (*p* < 0.001), higher NIHSS score at Emergency Department admission (*p* = 0.014), and longer time from stroke onset (*p* < 0.001) (Table 3).

On the log10 scale, each one-unit increase in eGFR was associated with a regression coefficient of −0.0107 (95% CI −0.0142 to −0.0072; *p* < 0.001), corresponding to a 2.4% decrease in absolute NfL concentration per mL/min/1.73 m^2^ increase. Each one-point increase in NIHSS was associated with a coefficient of 0.0151 (95% CI 0.0031 to 0.0271; *p* = 0.014), corresponding to an approximate 3.5% increase in NfL levels.

Time from stroke onset, modeled as a categorical variable (T0 as reference), was significantly associated with NfL levels. Compared with T0, log10 NfL increased by 0.332 (95% CI 0.280–0.384; *p* < 0.001) at T1 and by 0.561 (95% CI 0.474–0.647; *p* < 0.001) at T2 in the fully adjusted multivariable model, corresponding to increases of 115% and 263% in absolute NfL concentrations, respectively.

Table 4 summarizes the predicted NfL concentrations according to our model for different combinations of the three identified predictors.

As a secondary, clinically oriented analysis, we evaluated the association between KDIGO CKD stages and serum NfL concentrations. In the linear mixed-effects model, adjusted for time from stroke onset and NIHSS at admission, CKD stage was associated with serum NfL concentrations. Compared with G1, patients in G2 showed approximately 50.0% higher NfL levels (β = 0.176, 95% CI 0.0004–0.352; *p* = 0.050), whereas patients in G3 showed approximately 232.0% higher NfL levels (β = 0.521, 95% CI 0.285–0.757; *p* < 0.001).

### 3.4. Renal Function as Modifier of NfL Prognostic Value

Among patients with available 3-month follow-up and T2 NfL measurement (*n* = 62; 12 had mRS 3–6, of whom 2 died), the main effects of log10-transformed NfL at T2 and eGFR were not independently associated with an unfavorable 3-month outcome. The OR for mean-centered log10 NfL at T2 was 0.44 (95% CI 0.04–5.21; *p* = 0.516), while the OR for mean-centered eGFR was 0.80 (95% CI 0.43–1.46; *p* = 0.462). A statistically significant interaction between log10 NfL and baseline eGFR was observed (OR 0.22; 95% CI 0.07–0.73; *p* = 0.014), indicating that the association between NfL and functional outcome varied according to renal function.

Baseline NIHSS was independently associated with unfavorable outcome (OR 1.40 per point; 95% CI 1.11–1.78; *p* = 0.005), whereas age was not statistically significant (OR 1.09 per year; 95% CI 0.96–1.24; *p* = 0.175). Detailed model estimates are reported in Table 5.

**Figure 3:**
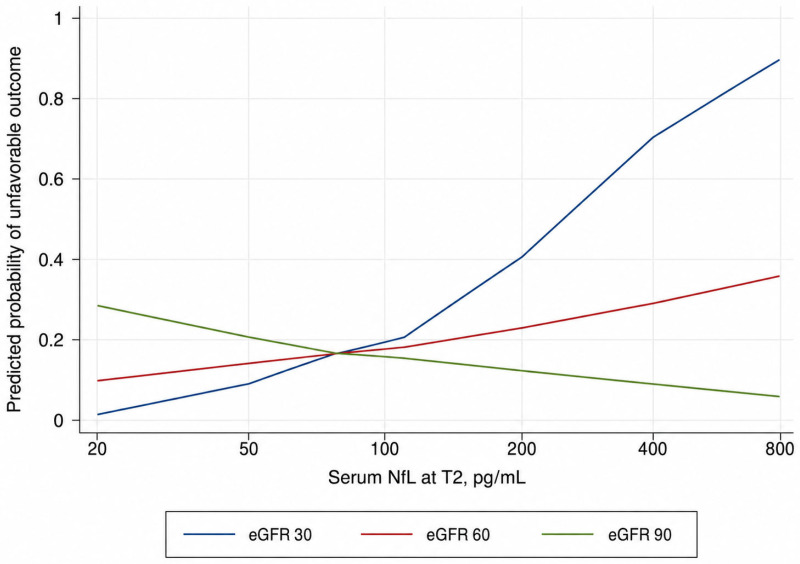
Predicted probability of unfavorable 3-month functional outcome according to serum NfL at T2 across eGFR strata.

Predicted-probability analysis showed that the association between serum NfL and unfavorable outcome was not constant across renal function levels. At lower eGFR values, increasing NfL was associated with a steeper increase in the predicted probability of unfavorable outcome, whereas this association was attenuated at intermediate eGFR values and minimal or unstable at higher eGFR values (Figure 3).

To further explore the prognostic role of serum NfL, we compared nested logistic regression models in the complete-case subgroup with available T2 NfL measurement and 3-month functional outcome. The base model included age, NIHSS at admission, and baseline eGFR. Adding log10-transformed NfL at T2 did not improve model fit (LR χ^2^(1) = 0.01; *p* = 0.9196). Conversely, the full model, including log10-transformed NfL at T2, eGFR, and their interaction significantly improved fit compared with the base clinical-renal model (LR χ^2^(2) = 6.90; *p* = 0.0317), and with the model including only the main effects of log10-transformed NfL and eGFR (LR χ^2^(1) = 6.89; *p* = 0.0087).

In exploratory ROC analysis, serum NfL at T2 alone showed moderate discrimination for unfavorable 3-month outcomes (AUC 0.696; 95% CI 0.507–0.884). The full model, including age, NIHSS, eGFR, log10-transformed NfL at T2, and the log10 NfL × eGFR interaction, showed the highest AUC (0.918; 95% CI 0.825–1.000), although the global comparison of ROC curves was not statistically significant (*p* = 0.259).

## 4. Discussion

Several studies have reported a relationship between renal function and NfL concentrations. For the first time, we have demonstrated an inverse correlation between renal function and serum NfL levels in a population of patients with acute ischemic stroke, a condition where this molecule is suddenly and massively released following acute neuronal damage. Quantitatively, each unit increase in eGFR corresponded to a 2.4% reduction in serum NfL concentrations in our study population. In addition, the secondary analysis based on KDIGO CKD stages showed a consistent gradient, with higher concentrations in patients with progressively reduced renal function.

These results align with previous data from patients during aging, and with neurodegenerative and neuroinflammatory diseases [20,21,22,23,24]. The proposed underlying mechanisms explaining the inverse relationship between eGFR and serum NfL concentrations include: (i) impaired NfL renal clearance due to reduced renal function, and (ii) the fact that antibodies used in NfL assays may detect not only the intact protein but also smaller fragments generated either by neuronal degradation or through NfL metabolism. These fragments are filtered at the glomerular level and may accumulate if eGFR is reduced [25].

Additionally, we observed that NfL levels are influenced by the time from stroke onset and by the extent of neurological deficit at ED admission. Serum NfL increased progressively across the first week after stroke, with significantly higher levels at both 5 ± 3 days and 7 ± 3 days compared with the hyperacute phase. These findings are in line with literature data that show a progressive serum NfL concentration increase after stroke, with a peak at approximately 7 days after onset [26].

As regards the positive association between NIHSS score at onset and NfL concentration, this finding is biologically plausible as NIHSS is a reliable marker of stroke severity and is associated with the extent of brain ischemic damage [27,28]. Being NfL a biomarker of axonal damage, higher concentrations in more severe strokes with larger lesions are expected. In our cohort, each additional point on the NIHSS scale was associated with a 3.5% increase in NfL levels.

Together, these findings strengthen the concept that NfL is a useful biomarker in the context of acute ischemic stroke, reflecting both the initial severity of neuronal injury and its temporal dynamics.

Importantly, beyond confirming these associations, our study provides evidence that renal function modifies the prognostic performance of NfL. The significant interaction between log10-transformed NfL and eGFR indicates that the association between NfL and 3-month functional outcome was not constant across renal function levels. Predicted-probability analysis showed a steeper increase in the predicted risk of unfavorable outcome with higher serum NfL in patients with lower eGFR. This association was attenuated at intermediate eGFR values and became minimal or unstable in patients with preserved renal function. Consistently, nested model comparison supported this interpretation, suggesting that the prognostic contribution of serum NfL may depend on renal function rather than reflecting a simple independent additive effect. Exploratory ROC analyses showed a numerically higher AUC for the model including the NfL × eGFR interaction, but this improvement was not statistically significant.

Overall, our findings support the concept that renal function may need to be taken into account when interpreting serum NfL as a prognostic biomarker in acute ischemic stroke. However, given the relatively small sample size, the limited number of events, and the uncertainty of estimates at extreme combinations of NfL and eGFR values, this interaction should be interpreted cautiously and considered hypothesis-generating. Confirmation in larger, multicenter cohorts will be necessary to determine the robustness and clinical relevance of this finding.

Beyond its influence on NfL concentrations, renal function is known to carry prognostic significance in the setting of acute ischemic stroke, which adds complexity to the interpretation of NfL as a prognostic biomarker. Reduced eGFR has been consistently identified as an independent predictor of unfavorable functional outcome and mortality after ischemic stroke. In a large multicenter Japanese registry (*n* = 10,395), eGFR < 45 mL/min/1.73 m^2^ was independently associated with unfavorable functional outcomes in patients with cardioembolic stroke (OR 1.30; 95% CI 1.01–1.69) and small vessel occlusion (OR 1.44; 95% CI 1.01–2.07) [29]. Similarly, in the Fukuoka Stroke Registry (*n* = 12,576), both decreased eGFR and proteinuria were independently associated with increased risks of stroke recurrence (HR 1.22; 95% CI 1.09–1.37 for eGFR < 45 vs. ≥60) and all-cause death (HR 1.45; 95% CI 1.33–1.57), even after adjustment for traditional cardiovascular risk factors [30]. A recent prospective study confirmed that higher eGFR was significantly associated with good 3-month functional outcomes after acute ischemic stroke (adjusted OR 2.634; 95% CI 1.207–5.748) [31].

The mechanisms underlying the independent prognostic impact of CKD on stroke outcomes extend beyond shared traditional risk factors: uremia-related factors including chronic inflammation, oxidative stress, abnormal calcium–phosphorus metabolism, and endothelial dysfunction contribute to worse stroke severity and outcomes [32]. CKD promotes arterial stiffness, vascular calcification, and a prothrombotic state, and is associated with a higher burden of silent cerebrovascular disease and pre-existing white matter injury [33,34]. These pathological processes may amplify the extent of ischemic brain damage and impair post-stroke recovery, independently of the initial lesion size.

Therefore, the observed interaction between NfL and eGFR in predicting functional outcome in our study may reflect not only the confounding effect of impaired NfL clearance but also a biological synergy. Patients with reduced renal function may exhibit higher NfL concentrations because of decreased clearance while simultaneously experiencing worse outcomes driven by CKD-related vascular and systemic pathology. Distinguishing between these two mechanisms remains a major challenge. Future studies should clarify the relative contribution of biomarker kinetics and renal dysfunction to improve the clinical translation of NfL as a surrogate endpoint in stroke research.

In this regard, the higher NfL concentrations associated with a steeper increase in the predicted probability of unfavorable outcome in patients with lower eGFR (Figure 3) are not consistent with a simple clearance-based confounding model. Such a model would predict a stronger NfL–outcome association in patients with preserved renal function. Instead, our findings suggest that CKD may act not only as a confounder of the NfL signal but also as an independent modifier of post-stroke outcome. This effect may be mediated by additional systemic mechanisms, including chronic inflammation, endothelial dysfunction, and reduced metabolic resilience, which are not directly captured by the biomarker itself. These considerations, together with the exploratory nature of the analysis, underscore the complexity of integrating NfL and renal function in prognostic models and the need for larger studies with quantitative neuroimaging data.

The identification of renal function as an independent predictor of serum NfL concentrations in this patient population adds an important dimension to its clinical interpretability and significance as a biomarker outcome measure. This interpretation is supported by the high prevalence of chronic kidney disease among patients with ischemic stroke. In a cohort of 232,236 patients aged ≥ 65 years, only 47.3% had an eGFR ≥ 60 mL/min/1.73 m^2^. The remaining patients showed varying degrees of renal impairment, while 2.8% were receiving dialysis [35]. Meta-analytic estimates suggest even higher figures, with eGFR < 60 mL/min/1.73 m^2^ present in ~30–56% of thrombolysis-treated patients and in ~16–42% of mechanical thrombectomy-treated cohorts [36].

These results suggest that renal function should be considered as a potential confounding variable when interpreting NfL levels in stroke research. Whether formal correction algorithms may improve the clinical utility of NfL remains to be established in larger, dedicated studies.

Our study has several limitations. First, the small sample size limited our ability to explore variability in renal function across subpopulations of patients, e.g., diabetics. In addition, the prognostic logistic regression analysis was performed in a smaller subgroup of patients with available T2 NfL measurement and 3-month follow-up. Therefore, despite the inclusion of a limited number of clinically relevant covariates, this model may be at risk of overfitting, and the observed NfL × eGFR interaction should be regarded as exploratory and hypothesis-generating. Second, although the study population was representative of the general spectrum of stroke etiologies, it consisted predominantly of Caucasian patients with mild-to-moderate stroke severity. Third, neuroimaging markers of infarct extent, such as ASPECTS or MRI-derived lesion volumes, were not included in the present analysis because of the heterogeneous distribution of stroke territories, the limited sample size available for subgroup analyses, and the lack of standardized MRI acquisition across all patients. Fourth, the median age of 68.3 years and the predominance of patients with mild-to-moderate stroke limit the generalizability of the results. This is also linked to the relatively high proportion of patients treated with intravenous thrombolysis and endovascular thrombectomy compared with general stroke patient cohorts, likely reflecting the tertiary-care setting and the study inclusion criteria, which selected patients presenting early after symptom onset, with preserved pre-stroke functional status and age ≤ 80 years. Fifth, we excluded patients older than 80 years and those with severe renal impairment (eGFR < 30 mL/min/1.73 m^2^), in line with eligibility criteria frequently adopted in interventional and biomarker-driven stroke trials [37,38]. This choice inevitably limits the generalizability of our findings. However, it was intended to improve the interpretability of serum NfL analyses by minimizing two major sources of baseline variability. In fact, in routine Stroke Unit populations, advanced chronic kidney disease accounts approximately for 5–6% of admissions, as shown in large multicenter cohorts such as Get With The Guidelines–Stroke (>230,000 ischemic strokes) [35,39], with similar proportions reported in European and Asian registries [40,41]. Therefore, even if included, our cohort would likely have contained only a few individuals with eGFR < 30 mL/min, an insufficient number for representative subgroup analyses but sufficient to introduce heterogeneity and confounding bias. Finally, detailed screening data for non-enrolled patients were not prospectively recorded, preventing the reconstruction of a complete patient-selection flowchart. This should be considered an additional limitation of the study.

## 5. Conclusions

Serum NfL concentrations after acute ischemic stroke are influenced by renal function. A reduction in eGFR is associated with an increase in NfL concentrations in the first week after acute ischemic stroke. Stroke severity and time-from-onset are additional independent predictors of NfL levels after acute ischemic stroke. These findings suggest the importance of considering renal function when interpreting NfL levels, given the high prevalence of chronic kidney disease in ischemic stroke patients.

## Figures and Tables

**Table 1:** Characteristics of the study population.

Variable	All Patients (*N* = 97)
Age, median (IQR)	68.3 (58.6–74.0)
Sex, female (%)	38 (39.2)
Ethnicity, *N* (%)	Caucasian: 95 (97.9)
	Black: 1 (1.0)
	Hispanic: 1 (1.0)
BMI, median (IQR)	25.1 (23.3–28.1)
Pre-stroke mRS, median (IQR)	0 (0–0)
NIHSS in ER, median (IQR)	5 (2–9)
NIHSS 0–4, *N* (%)	49 (50.5)
NIHSS 5–15, *N* (%)	39 (40.2)
NIHSS ≥16, *N* (%)	9 (9.3)
Intravenous thrombolysis, *N* (%)	40 (41.2)
Endovascular thrombectomy, *N* (%)	20 (20.6)
TOAST Classification (*N*, %)	
Large artery atherosclerosis	19 (19.6)
Cardioembolism	36 (37.1)
Small vessel occlusion	11 (11.3)
Other determined cause	8 (8.3)
Undetermined cause	23 (23.7)
Hypertension, *N* (%)	47 (48.5)
Atrial fibrillation, *N* (%)	12 (12.4)
Diabetes, *N* (%)	13 (13.4)
Dyslipidemia, *N* (%)	33 (34.0)
Smokers, *N* (%)	45 (46.4)
Carotid atherosclerotic disease, *N* (%)	18 (18.6)
Previous acute coronary syndrome, *N* (%)	5 (5.2)
Glycemia, mg/dL, median (IQR)	111.0 (98.0–127.0)
HbA1c %, median (IQR)	5.7 (5.4–6.1)
Total cholesterol, mg/dL, median (IQR)	173.0 (132.0–197.0)
HDL cholesterol, mg/dL, median (IQR)	48.6 (39.0–57.0)
LDL cholesterol, mg/dL, median (IQR)	105.0 (72.0–127.0)
Triglycerides, mg/dL, median (IQR)	93.5 (64.0–121.0)
C-reactive protein, mg/dL, median (IQR)	0.30 (0.11–0.69)
Serum creatinine, mg/dL, median (IQR)	0.85 (0.71–0.98)
eGFR_CKD-EPI, mL/min/1.73 m^2^ (median, IQR)	82.2 (66.0–89.9)

Abbreviations: CKD-EPI, Chronic Kidney Disease—Epidemiology Collaboration; BMI, body mass index; IQR, interquartile range; NIHSS, NIH stroke scale; SD, standard deviation.

**Table 3:** Results from the linear mixed-effects model for repeated measures of serum NfL levels with an unstructured residual variance–covariance structure and REML estimation. Dependent variable: log10-transformed serum neurofilament concentration. Model intercept (constant): 2.226 (95% CI: 1.924–2.527), *p* < 0.001. The left column reports the estimated percent change in absolute neurofilament concentration associated with a one-unit increase in each predictor variable.

Variable	Coefficient	95% CI	*p*-Value	Δ% Neurofilament
eGFR CKD-EPI (mL/min/1.73 m^2^)	−0.0106859	[−0.0141886; −0.0071832]	<0.001	−2.43%
Time from stroke (T1 vs. T0)	0.3321249	[0.2798674; 0.3843824]	<0.001	+114.9%
Time from stroke (T2 vs. T0)	0.560666	[0.4738519; 0.6474801]	<0.001	+263.4%
NIHSS in ED	0.0151182	[0.0031033; 0.0271332]	0.014	+3.54%

Abbreviations: CKD-EPI, Chronic Kidney Disease—Epidemiology Collaboration; ED, Emergency Department; eGFR, estimated Glomerular Filtration Rate; NIHSS, National Institute of Health Stroke Scale.

**Table 4:** Predicted marginal values of log10-transformed NfL levels and corresponding absolute serum concentrations, calculated for illustrative values of eGFR (30, 60, 90 mL/min/1.73 m^2^), NIHSS score (5, 10, 20), and time from stroke onset (T0, T1, T2).

eGFR mL/min/1.73 m^2^	NIHSS	Time from Stroke	log10_NfLT	Serum NfLT ^Ψ^ pg/mL
90	5	T0	1.34	21.9
		T1	1.672	47.1
		T2	1.9	79.5
	10	T0	1.415	26.0
		T1	1.747	55.8
		T2	1.976	94.7
	20	T0	1.566	36.8
		T1	1.898	79.1
		T2	2.127	134.0
60	5	T0	1.66	45.7
		T1	1.992	98.1
		T2	2.221	166.6
	10	T0	1.736	54.5
		T1	2.068	117.0
		T2	2.296	197.9
	20	T0	1.887	77.2
		T1	2.219	165.5
		T2	2.448	281.1
30	5	T0	1.981	95.8
		T1	2.313	205.4
		T2	2.541	347.9
	10	T0	2.056	113.9
		T1	2.388	244.2
		T2	2.617	414.0
	20	T0	2.208	161.6
		T1	2.54	346.8
		T2	2.768	585.8

^Ψ^ Derived from the inverse transformation of the logarithmic scale. Abbreviations: eGFR, estimated Glomerular Filtration Rate; NIHSS, National Institute of Health Stroke Scale; T0, blood sampling within 24 h from stroke onset; T1, blood sampling at 5 ± 3 days after stroke; T2, blood sampling at 7 ± 3 days after stroke.

**Table 5:** Multivariable logistic regression model for prediction of 3-month functional outcome. Dependent variable: unfavorable 3-month outcome (mRS 3–6). *N* = 62.

Variable	OR	95% CI	*p*-Value
log10 NfL at T2, mean-centered	0.44	[0.04; 5.21]	0.516
eGFR CKD-EPI, per 10 mL/min/1.73 m^2^ increase, mean-centered	0.80	[0.43; 1.46]	0.462
log10 NfL × eGFR/10	0.22	[0.07; 0.73]	0.014
Age (per year)	1.09	[0.96; 1.24]	0.175
NIHSS at admission (per point)	1.4	[1.11; 1.78]	0.005

Model fit: LR χ^2^(5) = 28.24; *p* < 0.001; pseudo-R^2^ = 0.4634. Abbreviations: eGFR, estimated Glomerular Filtration Rate; NIHSS, National Institutes of Health Stroke Scale; NfL, neurofilament light chain.

## Data Availability

Anonymized supporting data are available to qualified investigators from Zenodo at http://doi.org/10.5281/zenodo.17395289.
